# Automated Generation of Consistent Graph Models with First-Order Logic Theorem Provers

**DOI:** 10.1007/978-3-030-45234-6_22

**Published:** 2020-03-13

**Authors:** Aren A. Babikian, Oszkár Semeráth, Dániel Varró

**Affiliations:** 8 University of Paderborn, Paderborn, Germany; 9 ICREA, Open University of Catalonia, Barcelona, Spain; 10 McGill University, Montreal, Canada; 11 MTA-BME Lendület Cyber-Physical Systems Research Group, Budapest, Hungary; 12 Budapest University of Technology and Economics, Budapest, Hungary

**Keywords:** Domain-Specific Modeling Languages, Model Generation, Theorem Provers

## Abstract

The automated generation of graph models has become an enabler in several testing scenarios, including the testing of modeling environments used in the design of critical systems, or the synthesis of test contexts for autonomous vehicles. Those approaches rely on the automated construction of consistent graph models, where each model satisfies complex structural properties of the target domain captured in first-order logic predicates. In this paper, we propose a transformation technique to map such graph generation tasks to a problem consisting of first-order logic formulae, which can be solved by state-of-the-art TPTP-compliant theorem provers, producing valid graph models as outputs. We conducted performance measurements over all 73 theorem provers available in the TPTP library, and compared our approach with other solver-based approaches like Alloy and VIATRA Solver.

### 1 Introduction

**Motivation.** Synthetic graph models have been in use for many challenges of software engineering including the testing of object-oriented programs [18, 20], quality assurance of domain-specific languages [28], validation of model transformations [7] or performance benchmarks of model repositories [5]. In particular, various lines of research in model-driven engineering rely upon such graph models. Network science also heavily depends on the availability of graph models with designated distribution of nodes and edges.

Active research in automated graph model generation [10, 25, 30, 31] has been focusing on deriving graphs with desirable properties like consistency, diversity, scalability or realistic nature [37]. A particularly challenging task of domain-specific model generators is to ensure *consistency*, i.e. to guarantee that synthetic models are not only compliant with the metamodel of the domain, but they also satisfy additional well-formedness constraints captured in popular high-level languages like OCL or graph patterns.

**Problem statement.** Consistent graph generators frequently rely on back-end solvers by mapping model generation problems into logic formulae with different levels of expressiveness. For example, SAT-solvers are used by Kodkod [34] that map high-level languages to propositional logic, CSP-solvers are exploited in EMF2CSP [10], while SMT-solvers were applied in [12, 15, 28]. Consistent model generators may rely on custom search-based techniques [31], symbolic techniques [25] or custom decision procedures [9, 30] to improve scalability.

Automated theorem proving techniques have been developed within the automated reasoning community for decades with a wide range of supporting tools such as HOL [11] and Vampire [19]. In particular, first-order theorem provers have an extensive tool competition where each participating tool takes logic problems using a unified representation of first-order logic (FOL) formulae. This suggests that, despite not being designed for model generation, theorem provers may provide interesting results within the domain considering the success of other general-purpose approaches.

Interestingly, while theorem provers have been used in model-driven engineering to prove the consistency specifications (e.g. HOL-OCL [6], Maude, KeY), their performance has not been investigated in depth for model generation purposes. Since FOL theorem provers already have to face undecidability issues, they are typically optimized to quickly find inconsistencies in formal specifications, while generating a model as a proof of consistency may be less of a priority. As such, existing mappings to FOL formulae may not be reusable in their entirety when theorem provers are used for consistent model generation.

**Objectives.** In this paper, we aim to systematically investigate and evaluate the *use of first-order logic theorem provers for model generation purposes*. In particular, we present a mapping of domain specifications consisting of a *metamodel*, *well-formedness constraints* and an optional initial seed model to FOL formulae. Using the standard Thousands of Problems for Theorem Provers (TPTP) format for representing FOL formulae, we used 73 different theorem provers and solvers in a total of 87 different configurations to generate instance models of various size in the context of an industrial domain-specific modeling tool (Yakindu Statecharts) for a scalability evaluation of those solvers. Finally, model results can be transformed to instance models of the domain that can be opened in their native editor - although implementing this step turned out to be solver-specific.

**Added value.** While various back-end solvers have been used in related mappings, the integration and inclusion of an entire family of first-order logic theorem provers is a novel practical result. Furthermore, our paper provides the first evaluation of a wide range of theorem provers for model generation purposes. As an important technical side effect, thanks to a novel use of constants as object identifiers incorporated in the mapping to FOL formulae, we managed to significantly improve the scalability of the Z3 SMT-solver for model generation purposes compared to existing approaches [28, 32], which relied upon the native support of decision procedures in SMT-solvers.

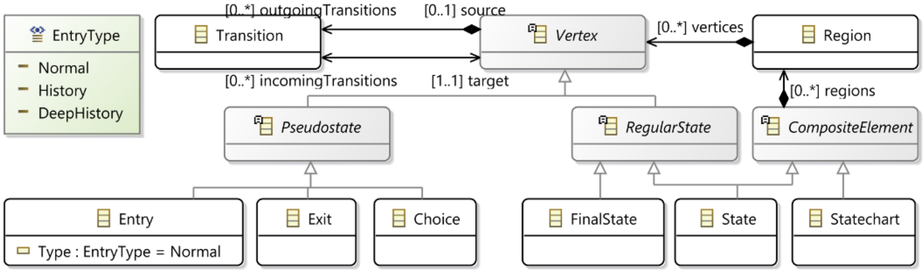

Fig. 1: Metamodel extract of Yakindu Statecharts

### 2 Preliminaries

The core concepts of domain-specific languages (DSL) and tools are illustrated in the context of Yakindu Statecharts [39], which is an industrial DSL for developing reactive, event-driven systems, and supports validation and code generation.

#### 2.1 Models and metamodels

In this paper we use EMF as a metamodeling technique which is widely used in the modeling community. Formally [28], an (EMF) metamodel defines a vocabulary $\Sigma = \{\mathtt{C}_1, \ldots, \mathtt{C}_n, \mathtt{R}_1, \ldots, \mathtt{R}_m, \mathtt{c}_1, \ldots, \mathtt{c}_o\}$, where a unary predicate symbol $\mathtt{C}_i$ is defined for each *EClass* and *EDataType* (like *EInteger* or *EEnums*), a binary predicate symbol $\mathtt{R}_j$ is derived for each *EReference* and *EAttribute*, and constant symbols $\mathtt{c}_k$ for *EEnum* literals.

*Example 1.* A simplified metamodel for Yakindu Statecharts is illustrated in Figure 1. A `Statechart` consists of `Regions`, which contain states (`Vertex`) and `Transitions`. The abstract state `Vertex` is further refined into `RegularStates` (like `State` or `FinalState`) and `PseudoStates` (like `Entry`, `Exit` or `Choice`). `Entry` states have a `Type` attribute of type `EntryType`.

Additionally, a metamodel also imposes several *structural constraints*:

1. *Type Hierarchy (TH)* expresses the correct combination of classes (e.g. if an object is an `Entry` then it must be a `Vertex`, but it cannot be a `Region`);
2. *Type Compliance (TC)* requires that for any relation $\mathtt{R}(o, t)$, its source and target objects $o$ and $t$ must have compliant types (e.g. the target of a reference `target` must be an instance of `Vertex`);
3. *Abstract (ABS)*: If a class is defined as abstract, it is not allowed to have direct instances (like `CompositeElement`);
4. *Multiplicity (MUL)* of structural features can be limited with upper and lower bound in the form of “lower..upper” (e.g. 1..1 for reference `target`);
5. *Inverse (INV)* states that two parallel references of opposite direction always occur in pairs (e.g. `outgoingTransitions` and `source`).
6. *Containment (CON)*: Instance models in EMF are expected to be arranged into a containment hierarchy, which is a directed tree along relations marked in the metamodel as containment (e.g., `vertices` or `outgoingTransitions`).

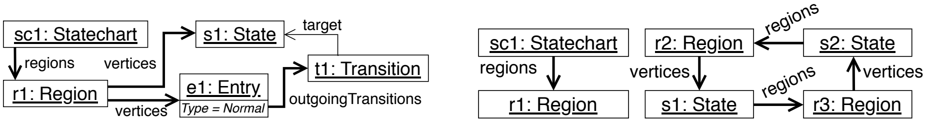

(a) Valid Yakindu instance model (b) Invalid, cyclic Yakindu instance model

Fig. 2: Sample Yakindu Statechart instance models

An *instance model* can be represented as a logic structure $M = \langle \mathcal{O}_M, \mathcal{I}_M \rangle$, where $\mathcal{O}_M$ is the finite set of objects, and $\mathcal{I}_M$ provides interpretation for all predicate symbols in $\Sigma$ as follows:

- The interpretation of a unary predicate symbol $\mathtt{C}_i$ is defined in accordance with the types of the EMF model: $\mathcal{I}_M(\mathtt{C}_i) : \mathcal{O}_M \to \{\mathtt{1}, \mathtt{0}\}$. An object $o \in \mathcal{O}_M$ is an instance of (more precisely, conforms to) a class $\mathtt{C}_i$ in a model $M$ if $\mathcal{I}_M(\mathtt{C}_i)(o) = \mathtt{1}$. It is possible for an object to conform to multiple types, e.g. in case of inheritance or abstract classes.
- The interpretation of a binary predicate symbol $\mathtt{R}_j$ is defined in accordance with the links in the EMF model: $\mathcal{I}_M(\mathtt{R}_j) : \mathcal{O}_M \times \mathcal{O}_M \to \{\mathtt{1}, \mathtt{0}\}$. There is a reference $\mathtt{R}_j$ between $o_1, o_2 \in \mathcal{O}_M$ in model $M$ if $\mathcal{I}_M(\mathtt{R}_j)(o_1, o_2) = \mathtt{1}$.
- The interpretation assigns each constant symbol $\mathtt{c}_k$: $\mathcal{I}_M : \mathtt{c}_k \to \mathcal{O}_M$.

*Example 2.* Figure 2a illustrates an instance model $M$ with objects $\mathcal{O}_M = \{sc1, r1, s1, t1, e1\}$. Classes of the object are added as labels (e.g. label sc1: Statechart denotes $\mathcal{I}_M(\mathtt{Statechart})(sc1) = \mathtt{1}$), attribute values are illustrated as attribute=value labels (e.g. Type = Normal as $\mathcal{I}_M(\mathtt{Type})(e1, \mathtt{Normal}) = \mathtt{1}$), and reference predicates as labelled edges (e.g. regions edge from $sc1$ to $r1$ as $\mathcal{I}_M(\mathtt{regions})(sc1, r1) = \mathtt{1}$).

### 2.2 Model predicates and Well-formedness constraints

In many industrial modeling tools, domain-specific WF constraints are defined by *error predicates* captured either as OCL constraints [24] or as graph patterns [35]. A major practical subclass of such constraints can be formalized using first-order logic predicates [28].

A graph predicate $\varphi$ is defined inductively over a vocabulary $\Sigma$ of a metamodel and an infinite set of (object) variables $\{v_1, v_2, \ldots\}$ and the constant symbols as seen in Figure 3a. A graph predicate $\varphi$ with free variables $param = \{v_1, \ldots, v_n\}$ can be evaluated over a model $M$ with variable binding $Z : param \to \mathcal{O}_M$ (denoted with $[\![\varphi(v_1, \ldots, v_n)]\!]^M_Z$) using the rules of Figure 3b.

Therefore, if a domain defines error patterns $\varphi_1, \ldots, \varphi_n$, a model is considered consistent (valid), if it does not satisfy any error predicates $\varphi_i(v_1, \ldots, v_m)$ $(1 \leq i \leq n)$, i.e. $\forall v_1, \ldots, v_m : \neg\varphi_i(v_1, \ldots, v_m)$. Since a formalization of these structural restrictions as WF constraints is provided in [28], the predicate language of Figure 3b can uniformly be used for both kinds of structural constraints.

*Example 3.* Figure 4 illustrates three graph patterns defined in both graphical and textual syntax. Pattern `transition(t,src,trg)` defines a relation

<table>
<tr><th></th><th>Logic Syntax</th><th>TPTP Syntax</th><th></th></tr>
<tr><td>$\varphi :=$</td><td>$c$</td><td>c</td><td>constant</td></tr>
<tr><td>$\mid$</td><td>$\texttt{C}(v)$</td><td>C(v)</td><td>type predicate</td></tr>
<tr><td>$\mid$</td><td>$\texttt{R}(v_1, v_2)$</td><td>R(v1,v2)</td><td>reference predicate</td></tr>
<tr><td>$\mid$</td><td>$v_1 = v_2$</td><td>v1=v2</td><td>equivalence</td></tr>
<tr><td>$\mid$</td><td>$dist(v_1, ..., v_n)$</td><td>v1!=v2 &...&vn-1!=vn</td><td>n-ary inequality (distinctness)</td></tr>
<tr><td>$\mid$</td><td>$\neg\varphi \mid \varphi_1 \wedge \varphi_2 \mid \varphi_1 \vee \varphi_2$</td><td>~ p | p1&p2 | p1|p2</td><td>logic connectives</td></tr>
<tr><td>$\mid$</td><td>$\exists v : \varphi \mid \forall v : \varphi$</td><td>?[v]:p | ![v]:p</td><td>quantified expression</td></tr>
</table>

(a) Syntax of graph predicates

$$
\begin{aligned}
[\![c]\!]^M_Z &:= \mathcal{I}_M(c) \\
[\![\texttt{C}(v)]\!]^M_Z &:= \mathcal{I}_M(\texttt{C})(Z(v)) \\
[\![\texttt{R}(v_1, v_2)]\!]^M_Z &:= \mathcal{I}_M(\texttt{R})(Z(v_1), Z(v_2)) \\
[\![v_1 = v_2]\!]^M_Z &:= Z(v_1) = Z(v_2) \\
[\![\neg\varphi]\!]^M_Z &:= 1 - [\![\varphi]\!]^M_Z
\end{aligned}
\qquad
\begin{aligned}
[\![\varphi_1 \wedge \varphi_2]\!]^M_Z &:= min([\![\varphi_1]\!]^M_Z, [\![\varphi_2]\!]^M_Z) \\
[\![\varphi_1 \vee \varphi_2]\!]^M_Z &:= max([\![\varphi_1]\!]^M_Z, [\![\varphi_2]\!]^M_Z) \\
[\![\exists v : \varphi]\!]^M_Z &:= max_{o \in \mathcal{O}_M}\{[\![\varphi_1]\!]^M_{Z, v \mapsto o}\} \\
[\![\forall v : \varphi]\!]^M_Z &:= min_{o \in \mathcal{O}_M}\{[\![\varphi_1]\!]^M_{Z, v \mapsto o}\}
\end{aligned}
$$

(b) Semantic rules for graph predicates

Fig. 3: Syntax and semantics for graph predicates

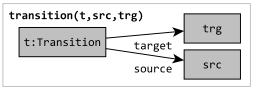

```
pattern transition(t,src,trg) {
  Transition.source(t,src);
  Transition.target(t,trg); }
```

$$transition(t, src, trg) = \texttt{source}(t, src) \wedge \texttt{target}(t, trg)$$

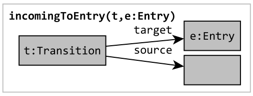

```
@Constraint
pattern incomingToEntry(t, e:Entry) {
  find transition(t,_,e); }
```

$$ite(t, e) = \exists s : transition(t, s, e) \wedge \texttt{Entry}(e)$$

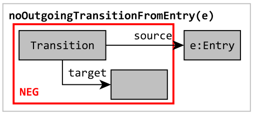

```
@Constraint
pattern noOutgoing(e:Entry) {
  neg find transition(_,e,_); }
```

$$no(e) = \forall t, trg : \neg transition(t, e, trg) \wedge \texttt{Entry}(e)$$

Fig. 4: Example graph patterns defined with graphical and VIATRA syntax between two `Vertices` which are connected via a `Transition` using `source` and `target` references. Reusing this pattern, two WF constraints are defined concerning `Entry` states: if any of them has a match, then the model is malformed. First, `incomingToEntry(t, e)` selects invalid `Transitions` that are leading to an `Entry` (by reusing the previously defined `transition` pattern). Next, `noOutgoingTransitionFromEntry(e)` matches to `Entry` states that does not have any outgoing `Transition` (by negatively using `transition` pattern).

#### 2.3 First-Order Logic Theorem Provers

Our approach to model generation involves using a back-end FOL theorem prover to generate finite models according to input constraints. The theorem prover is treated as a black-box component in our model generation workflow, thus it takes input formulae and generates an output formula. Logic formulae are given using the TPTP Syntax [33] as it is a standard within the theorem prover community.

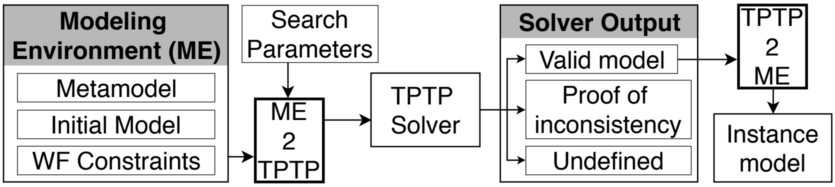

Fig. 5: Overview of our model generation approach

The TPTP syntax defines multiple forms of logic formulae, such as Full First-order Form (FOF) and Typed Higher-order Form (THF). Our mapping derives FOF formulae defined by a subsyntax that can handle standard FOL statements. This is sufficient for modeling most aspects of EMF and WF constraints. Omitted aspects include containment cycle avoidance and numeric attributes

Regarding the output of TPTP-compliant theorem provers, there does not seem to be a standard. Provers may output FOF formulae, other TPTP formulae, or TPTP non-compliant formulae. This is not surprising, as many TPTP-compliant solvers also handle various other syntaxes. As a result, in order to interpret the output of TPTP-compliant provers, one must create a custom parser for each prover, which is laborious. However, despite syntactic differences, prover outputs are structurally similar: in most cases, the output contains a list of graph nodes, where each node is associated to corresponding types and graph edges.

### 3 Overview of the Approach

Our approach (summarized in Figure 5) aims to generate graph models that are consistent with respect to WF constraints of a domain-specific *modeling environment* using theorem provers as back-end solvers. For this purpose, we map the high-level specifications of the input DSL into equivalent FOL formulae written in TPTP-compliant syntax [33]. We implement our approach as part of the VIATRA Model Generation Framework [1].

The specification of the DSL (or modeling environment) consists of a *metamodel* specified in EMF augmented with *well-formedness constraints* captured by model queries (using the VIATRA framework [36]). Additionally, our generator can take an optional *initial instance model* that acts as a seed for model generation. Our model generation framework can also take various *search parameters* such as type scope (requested size) and containment cycle avoidance specifications as input to guide model generation towards desired characteristics.

The input modeling environment and the search parameters are mapped to FOL formulae using the novel *ME2TPTP* model-to-text transformation detailed in section 4. The FOL-formula is then fed into a TPTP-compliant theorem prover (*TPTP Solver*). The solver may output a *valid model* if all input constraints are satisfiable. In this case, the output is transformed into a domain-compliant *instance model* through a *TPTP2ME* backwards mapping. Otherwise, if input constraints are inconsistent, the solver can either identify its *inconsistency*, or provides an *undefined* output (if it cannot decide by its decision procedures or due to lack of computational resources).

Our approach is designed to generate a finite model rather than a finite counterexample of the input specifications. Such a task is facilitated by including size requirements for the desired model a priori. However, if size requirements are not provided, the theorem prover could easily check for inconsistencies in the input formulae due to the small-model theorem [14].

In addition to generating graph models from scratch, our approach is also capable of completing initial seed models. An initial model may be inconsistent (i.e. it may violate some metamodel or WF constraints), thus it is the task of the TPTP solver to extend the input model into a consistent instance model. Another use case is to validate the consistency of DSLs and modeling environments [16, 28]. Our approach is capable of detecting when constraints derived from a modeling environment are contradictory with each other. In this case, our approach can prove the unsatisfiability of the input constraints.

### 4 From Domain-Specific Languages to First-Order Logic

We discuss how the various components of a modeling language are mapped into a set $\varphi = \varphi^{MM} \wedge \varphi^{IM} \wedge \varphi^{WF}$ of TPTP-compliant FOL formulae. The formula $\varphi^{MM}$ is derived from the metamodel types (in section 4.1) and relations (in section 4.2) , as defined in section 2.1 , along with additional constraints and search parameters. $\varphi^{IM}$ describes the mapping for initial instance models (in section 4.3). Finally, $\varphi^{WF}$ describes how additional WF constraints defined as VIATRA queries are mapped into FOL formulae (in section 4.4). All components of our mapping with the exception of lower multiplicities and WF constraints output Essentially Propositional Logic formulae. Proof systems for such formulae [23] do exist, but cannot be fully exploited on the output of our mapping.

#### 4.1 Mapping Types in the Metamodel

The various types in the input EMF metamodel are mapped to FOL formulae as described below.

**Objects:** A key idea in our mapping is that we use FOL constants (instead of other data types such as TPTP distinct objects) to represent the generated graph nodes. Constants are preferred due to their compatibility with our presented encoding (distinct objects cannot be used as arguments for FOL predicates).

These constants are separated into two categories: first, nodes defined prior to theorem proving are denoted with a set of constant symbols $Obj^O = \{old_1, \ldots, old_n\}$. This set includes known objects such as enum literals and elements of the initial partial model. Additionally, the logic solver will add new objects to the generated model, some of which are denoted with constant symbols $Obj^N = \{new_1, \ldots, new_m\}$. We also introduce a unary predicate $object(o)$ that selects all nodes of the graph model (including attribute values, enum literals and objects). The $object(o)$ predicate holds for all constants $o$ in $Obj^O$ and for some in $Obj^N$.

EMF

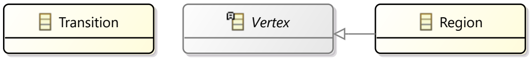

FOL

**TH1:** $\forall o : object(o) \Rightarrow$

| | $\texttt{Transition}$ | $\texttt{Vertex}$ | $\texttt{Region}$ |
|---|---|---|---|
| $\texttt{Transition}$ | ( $\texttt{Transition}(o) \wedge$ | $\neg\texttt{Vertex}(o) \wedge$ | $\neg\texttt{Region}(o)$ )$\vee$ |
| $\texttt{Region}$ | ( $\neg\texttt{Transition}(o) \wedge$ | $\texttt{Vertex}(o) \wedge$ | $\texttt{Region}(o)$ ) |

**TH2:** $\forall o : \neg object(o) \Rightarrow$
$\neg\texttt{Transition}(o) \wedge \neg\texttt{Vertex}(o) \wedge \neg\texttt{Region}(o))$

Fig. 6: Mapping type hierarchy

**Type Hierarchy (TH):** To handle complex generalization relations (e.g multiple inheritance) in the type hierarchy, we introduce formulae to control the potential combinations of the type predicates. For this purpose, we map each *EClass* of the input metamodel to a FOL predicate $\texttt{C}_i(o)$. A sample mapping is shown in Figure 6 for an extract of the domain metamodel.

To express the *mutual exclusiveness* of (non-abstract) classes in the type hierarchy, we construct a formula $d_0 = \bigvee_{\texttt{C}_i \in s_{na}} t_i(\texttt{C}_i)$ in disjunctive normal form (DNF) for the set $s_{na}$ of all non-abstract classes in the metamodel. For each non-abstract type $\texttt{C}_i$, a conjunction $t_i(\texttt{C}_i)$ is created for all class predicates such that a predicate $\texttt{C}_j$ is positive if and only if it is a member of set $s(\texttt{C}_i)$ containing $\texttt{C}_i$ and its superclasses, formally $t_i(\texttt{C}_i) = \bigwedge_{\texttt{C}_j \in s(\texttt{C}_i)} \texttt{C}_j(o) \wedge \bigwedge_{\texttt{C}_j \notin s(\texttt{C}_i)} \neg\texttt{C}_j(o)$. We must ensure that any constant satisfying the $object(o)$ predicate also satisfies the type hierarchy described in $d_0$. Thus, we generate the following FOL formula: $\varphi^{MM}_{\mathbf{TH1}} = \forall o : object(o) \Rightarrow d_0$. This is a *filtered-types* approach to type hierarchy transformations used in the context of Object-Relation Mapping [17].

We also generate a formula to handle the negative case for the *object* predicate. We specify that any constants $o$ that is not compliant with the $object(o)$ predicate must not be an instance of any class in the metamodel. Formally, the negation of $object(o)$ implies a conjunction $t_{no}$ of the negations of all class predicates $\texttt{C}_i$ in the metamodel (MM): $t_{no} = \bigwedge_{\texttt{C}_i \in MM} \neg\texttt{C}_i$. The generated FOL formula is as follows: $\varphi^{MM}_{\mathbf{TH2}} = \forall o : \neg object(o) \Rightarrow t_{no}$.

**Enumerations and Literals (EN)** Mapping for enumerations is carried out similarly to that of types. A unary predicate is created for each enum class $\texttt{E}_i(o)$ in the input metamodel, and a distinct unary predicate $\texttt{l}_i(o)$ is created for each literal of the enum class. The mapping of an enum class creates a disjunction $d_1 = \bigvee_{\texttt{l}_i} t_i(\texttt{l}_i)$. For each literal $\texttt{l}_i$, a conjunction $t_i$ is created, where only the predicate corresponding to $\texttt{l}_i$ is positive and all others are negative, formally $t_i(\texttt{l}_i) = \texttt{l}_i(o) \wedge \bigwedge_{\texttt{l}_j \neq \texttt{l}_i} \neg\texttt{l}_j(o)$. To ensure that generated enum instances are part of the output model and that each literal is unique, a FOL constraint is generated for each enum class stating that objects satisfy the corresponding predicate $\texttt{E}_i$ if and only if they also satisfy the $object(o)$ predicate and the disjunction $d_1$:

$$\varphi^{MM}_{\mathbf{EN1}} = \forall o : \texttt{E}_i(o) \Leftrightarrow object(o) \wedge \bigvee_{\texttt{l}_i} \left( \texttt{l}_i(o) \wedge \bigwedge_{\texttt{l}_j \neq \texttt{l}_i} \neg\texttt{l}_j(o) \right)$$

**EN1:** $\forall o : \texttt{EntryType}(o) \Leftrightarrow (object(o) \wedge ((\texttt{Normal}(o) \wedge \neg\texttt{History}(o)) \vee (\neg\texttt{Normal}(o) \wedge \texttt{History}(o))))$

**EN2-N:** $\forall o : (o = eo_1 \Leftrightarrow \texttt{Normal}(o))$

**EN2-H:** $\forall o : (o = eo_2 \Leftrightarrow \texttt{History}(o))$

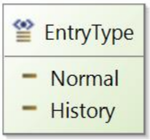

Fig. 7: Mapping enumerations

Each enum literal is also transformed into an individual FOL constraint that instantiates a constant $eo_i$ to define an enum object for each $\texttt{l}_i$ that is associated with $\texttt{E}_i$. The generated FOL constraint ensures that the output model contains a constant $eo_i$ corresponding to each enum literal: $\varphi^{MM}_{\mathbf{EN2}} = \forall o : (o = eo_i \Leftrightarrow \texttt{l}_i(o))$.

*Example 4.* To better understand this mapping, we consider the `EntryType` enum in Figure 1. We omit the `DeepHistory` literal for the sake of conciseness. This enum is mapped into the 3 FOL statements shown in Figure 7.

**Model Scope:** Our mapping also allows for users to specify a scope (size) for the generated model as a search parameter. A scope may contain an upper bound $u$ and a lower bound $l$ for the number of generated objects in the output model. For an upper bound specification $u$, we define $Obj^N = \{new_1, \ldots, new_{u-|Obj^O|}\}$, where $Obj^O$ is the set of nodes defined prior to theorem proving. If $u - |Obj^O|$ is negative then the problem is surely inconsistent. We then generate a FOL expression which specifies that any constant $o$ satisfying $object(o)$ must be contained in either $Obj^O$ or $Obj^N$, to ensure that the theorem prover does not generate any further constants (that satisfy $object(o)$) as part of the output finite model.

$$\varphi^{MM}_{\mathbf{MUB}} = \forall o : object(o) \Rightarrow \left( \bigvee_{old_i \in Obj^O} (o = old_i) \vee \bigvee_{new_i \in Obj^N} (o = new_i) \right)$$

For a lower bound specification $l$, we define $m' = l - |Obj^O|$ and we create a set $Obj^N_{lb} \subseteq Obj^N$ containing $m'$ constants that are also in $Obj^N$. In the case where $Obj^N$ is not defined (an upper bound value has not been specified), we define $Obj^N_{lb} = \{new_1, \ldots, new_{l-|Obj^O|}\}$. We then generate a FOL formula to specify that any object $o$ that is either in $Obj^O$ or in $Obj^N_{lb}$ must also satisfy $object(o)$ to ensure that these constants are part of the output finite model:

$$\varphi^{MM}_{\mathbf{MLB}} = \forall o : \left( \bigvee_{old_i \in Obj^O} (o = old_i) \vee \bigvee_{new_i \in Obj^N_{lb}} (o = new_i) \right) \Rightarrow object(o)$$

*Example 5.* To generate a model that contains from 4 to 6 objects, 2 of which are already defined (e.g. enum literals), the following FOL statements are derived:

**MUB:** $\forall o : object(o) \Rightarrow ((o = old_1) \vee (o = old_2) \vee (o = new_1) \vee (o = new_3) \vee (o = new_4) \vee (o = new_2))$

**MLB:** $\forall o : ((o = old_1) \vee (o = old_2) \vee (o = new_1) \vee (o = new_2)) \Rightarrow object(o)$

EMF

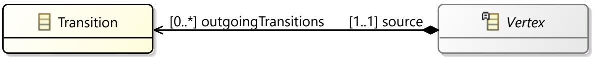

FOL

**TC:** $\forall v, t : (\texttt{outgoingTransitions}(v,t) \Rightarrow (\texttt{Vertex}(v) \land \texttt{Transition}(t))$

$\forall v, t : (\texttt{source}(t,v) \Rightarrow (\texttt{Transition}(t) \land \texttt{Vertex}(v))$

**MUL:** $\forall t : \texttt{Transition}(t) \Rightarrow \exists v : \texttt{source}(t,v)$

**MUU:** $\forall t, v_1, v_2 : (\texttt{source}(t,v_1) \land \texttt{source}(t,v_2)) \Rightarrow v_1 = v_2$

**INV:** $\forall v, t : \texttt{source}(t,v) \Leftrightarrow \texttt{outgoingTransitions}(v,t)$

**CO1:** $\forall v, t : contains(v,t) \Leftrightarrow \texttt{outgoingTransitions}(v,t)$

Fig. 8: Mapping relations

**Type Scope:** A scope may be specified for each particular type $\texttt{C}$. In the case of an upper bound $u_t$, we define a set $Obj^N_{ut}$ such that $u_t = |Obj^N_{ut}|$. If a model upper bound has been defined, then $Obj^N_{ut} \subseteq Obj^N$ holds, and we specify that any constant $o$ satisfying $object(o)$ and $\texttt{C}_i(o)$ must be contained in $Obj^N_{ut}$:

$$\varphi^{MM}_{\mathbf{TUB}} = \forall o : (object(o) \land \texttt{C}_i(o)) \Rightarrow \bigvee_{new_i \in Obj^N_{ut}} (o = new_i)$$

In case of a lower bound $l_t$, we select a set $Obj^N_{lt} \subseteq Obj^N_{ut}$ (if $Obj^N_{ut}$ is defined) such that $l_t = |Obj^N_{lt}|$. We then generate a FOL expression which specifies that all constants in $Obj^N_{lt}$ must also satisfy $object(o)$ and $\texttt{C}_i(o)$:

$$\varphi^{MM}_{\mathbf{TLB}} = \forall o : \bigvee_{new_i \in Obj^N_{lt}} (o = new_i) \Rightarrow (object(o) \land \texttt{C}_i(o))$$

**Uniqueness:** For every model object mapped to a FOL constant $c_i$, we must generate formulae to ensure that it is distinct from other objects. These formulae are only generated in the case where a scope is defined. Assuming that an ordering is defined for all $n$ constants $c_i$, we generate $n-1$ FOL constraints with increasing value of $j < n$: $\varphi^{MM}_{\mathbf{Un}}(j,n) = \bigwedge^n_{c_i : i=j+1} c_j \neq c_i$.

### 4.2 Mapping Relations Between Metamodel Types

Once type-related constraints are mapped into FOL formulae, relations between these types are mapped as binary predicates.

**Type Compliance (TC)** Relations between classes and class attributes are mapped into FOL in the same way (see section 2). Each relation and attribute is mapped to a FOL predicate $\texttt{R}_i(o_1, o_2)$. When mapping relations, we must ensure that the endpoint objects are type-compliant with the metamodel: for each $\texttt{R}_i(o_1, o_2)$ that points from a class $\texttt{C}_1$ to a type $\texttt{C}_2$, we generate a formula

$$\varphi^{MM}_{\mathbf{TC}} = \forall o_1, o_2 : \texttt{R}_i(o_1, o_2) \Rightarrow (\texttt{C}_1(o_1) \land \texttt{C}_2(o_2)).$$

Note that for the purpose of this specific mapping, inverse relations are considered as two separate unidirectional relations. Figure 8 contains an example of such a case, with the corresponding TC mapping.

**Multiplicities (MUL)** As the multiplicity of a unidirectional relation has a lower and an upper bound, at most two FOL formulae will be generated. Lower multiplicities of 0 and upper multiplicities of $*$ do not generate any formulae.

*Lower Multiplicity :* Consider the relation $\mathtt{R}_i(a,b)$ from $\mathtt{C}_i(a)$ to $\mathtt{C}_j(b)$ which has a lower multiplicity $m \neq 0$. We generate the constraint that for all objects $a$ of type $\mathtt{C}_i(a)$, there must exist at least $m$ unique constants $b_0 \dots b_m$ connected to $\mathtt{C}_i(a)$ through a $\mathtt{R}_i(a,b_i)$ relation. The generated FOL constraint is:

$$\varphi^{MM}_{\mathbf{MUL}} = \forall a : \left(\mathtt{C}_i(a) \Rightarrow \left(\exists b_0 \dots b_m : \left(\bigwedge_{b_i : i=0}^{m} \mathtt{R}_i(a,b_i)\right) \wedge \mathit{distinct}(b_0 \dots b_m)\right)\right)$$

*Upper Multiplicity :* Given the relation $\mathtt{R}_i(a,b)$ introduced previously, let us consider an upper multiplicity of $n \neq *$. We generate the constraint that if there are $n+1$ objects $b_0 \dots b_{n+1}$ connected to an object $a$ through $\mathtt{R}_i(a,b_i)$ relations, then there are at least 2 identical $b_i$ constants among $b_0 \dots b_{n+1}$. This means that $b_0 \dots b_{n+1}$ are not pairwise distinct, formally $\neg \mathit{distinct}(b_0 \dots b_{n+1})$.

$$\varphi^{MM}_{\mathbf{MUU}} = \forall a, b_0 \dots b_{n+1} : \left(\bigwedge_{b_i : i=0}^{n+1} \mathtt{R}_i(a,b_i)\right) \Rightarrow \neg \mathit{distinct}(b_0 \dots b_{n+1})$$

Multiplicity formulae derived from a relation in Figure 1 are shown in Figure 8. Note the asymmetric nature of the two formulae: lower multiplicities are more difficult to satisfy for the prover as that might introduce an infinite model.

**Inverse Relations (INV)** As mentioned earlier, we consider inverse relations as two separate (unidirectional) relations. The bidirectional nature of such relations implies that both of their corresponding unidirectional relations cannot exist without each other. Thus, we must ensure that for two objects $a$ and $b$ are connected by inverse relations $\mathtt{R}_i(a,b)$ and $\mathtt{R}_j(b,a)$ simultaneously: $\varphi^{MM}_{\mathbf{INV}} = \forall a, b : \mathtt{R}_i(a,b) \Leftrightarrow \mathtt{R}_j(b,a)$. An example can be seen in Figure 8.

**Containment Hierarchy (CON)** Containment hierarchy is enforced by the following constraints (see Figure 8 for examples):

– *Union of containment edges:* We first define a disjunction $\mathit{contains}(o_1,o_2)$ of all containment relations $\mathtt{R}_{c-i}(o_1,o_2)$ in the metamodel. The generated FOL formula is $\varphi^{MM}_{\mathbf{CO1}} = \forall o_1, o_2 : \mathit{contains}(o_1,o_2) \Leftrightarrow \bigvee_{\mathtt{R}_{c-i}} \mathtt{R}_{c-i}(o_1,o_2)$.
– *Existence of a unique root constant:* We define a unique constant `root` as an object that is not contained: $\varphi^{MM}_{\mathbf{CO2}} = \forall r, o : (r = \mathtt{root} \Leftrightarrow \neg \mathit{contains}(o,r))$.
– *Container Object:* We must ensure that every non-root object in the generated model is contained by another object. Thus, any constant $o$ that satisfies $\mathit{object}(o)$ is either the root constant `root` or is contained by another constant. Formally, $\varphi^{MM}_{\mathbf{CO3}} = \forall o : \mathit{object}(o) \Rightarrow (o = \mathtt{root} \vee \exists p : \mathit{contains}(p,o))$.
– *Single Container:* We must also ensure that any constant $o$ is contained by at most one other constant. Thus, if $o$ is contained by two constants $p_1$ and $p_2$, then $p_1$ and $p_2$ are identical. Formally, $\varphi^{MM}_{\mathbf{CO4}} = \forall o, p_1, p_2 : (\mathit{contains}(p_1,o) \wedge \mathit{contains}(p_2,o)) \Rightarrow (p_1 = p_2)$.

EMF

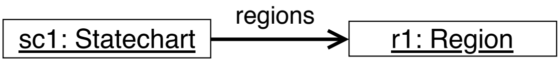

FOL

**obj→const:** $sc1 \mapsto old_1, r1 \mapsto old_2$

$\forall o : \texttt{SChart}(o) \Leftrightarrow (\texttt{SChart}^O(o) \wedge \neg\texttt{SChart}^N(o)) \vee (\neg\texttt{SChart}^O(o) \wedge \texttt{SChart}^N(o))$

$\forall o : \texttt{Region}(o) \Leftrightarrow (\texttt{Region}^O(o) \wedge \neg\texttt{Region}^N(o)) \vee (\neg\texttt{Region}^O(o) \wedge \texttt{Region}^N(o))$

**PS-C:** $\forall o : \texttt{Statechart}^O(o) \Leftrightarrow o = old_1, \quad \forall o : \texttt{Region}^O(o) \Leftrightarrow o = old_2$

**PS-R:** $\texttt{regions}(old_1, old_2)$

Fig. 9: Mapping instance models

**Avoidance of Cyclic Containment (CYC)** Unfortunately, FOL is not expressive enough to capture formulae required to avoid cyclic containment relations (an example is shown in Figure 2b) in the output models. Therefore, we generate approximated constraints to avoid cycles up to length $n$ given as an input parameter. For that purpose, we derive separate formulae for each length $x$ (with $0 < x \leq n$) using the $contains(o_1, o_2)$ predicate defined in $\varphi^{MM}_{\mathbf{CO1}}$. Formally, $\varphi^{MM}_{\mathbf{CYC}}(x) = \neg\exists o_1 \dots o_x : \left(\bigwedge_{i=0}^{x-1} contains(o_i, o_{i+1})\right) \wedge contains(o_x, o_0)$.

#### 4.3 Instance model mapping

When mapping an instance model $P = \langle \mathcal{O}_P, \mathcal{I}_P \rangle$ as a partial snapshot, we transform its objects $\mathcal{O}_P = \{o_1, \dots, o_n\}$ to a set of constants $Const_P = \{old_1, \dots, old_n\}$ while maintaining a trace map $t : \mathcal{O}_P \rightarrow Const_P$. Additionally, all classes $\texttt{C}$ which have an instance in the instance model are split into two categories: $\texttt{C}^O$ and $\texttt{C}^N$ that differentiate the old (i.e. $old_1, \dots, old_n$) and new objects (generated by the solver). Finally, if a class predicate $\texttt{C}_i$ is true in the partial model $\mathcal{I}_P(\texttt{C}_i)(o) = \texttt{1}$, then it must be true in the generated model too, which is enforced by formula $\texttt{C}_i^O(t(o))$. Similarly, if a reference predicate $\texttt{R}_j$ is true in the partial model $\mathcal{I}_M(\texttt{R}_j)(o_1, o_2) = \texttt{1}$, then it also must be true in the generated model, which is enforced by formula $\texttt{R}_j(t(o_1), t(o_2))$.

A sample generated FOL formulae for an instance model is shown in Figure 9.

#### 4.4 Mapping additional constraints

The modeling environment of our approach may contain additional FOL patterns and WF constraints defined in the Viatra Query Language (VQL). The header of each VQL pattern taking $n$ parameters as input is mapped to a predicate $\texttt{ph}_i(v_1 \dots v_n)$. The pattern body is mapped into a FOL statement $\varphi_{pci}(v_1 \dots v_n)$ according to its FOL content such that if a set of $n$ variables satisfy the associated pattern header predicate, it must also satisfy the specifications described in $\varphi_{pci}(v_1 \dots v_n)$: $\varphi^{WF}_{\mathbf{WF1}} = \forall v_1 \dots v_n : \texttt{ph}_i(v_1 \dots v_n) \Rightarrow \varphi_{pci}(v_1 \dots v_n)$.

For patterns that are specified as WF constraint, an additional FOL formula is generated to ensure that such patterns does not matching in the generated model. Structurally, the corresponding FOL formula checks that no objects $v_1 \dots v_n$ satisfies the condition of the pattern: $\varphi^{WF}_{\mathbf{WF2}} = \forall v_1 \dots v_n : \neg\texttt{ph}_i(v_1 \dots v_n)$. Figure 10 shows the mapping for patterns specified in Figure 4.

| | | |
|---|---|---|
| VQL | `pattern transition(t,src,trg){ Transition.source(t,src); Transition.target(t,trg); }` | `@Constraint pattern incomingToEntry(t, e:Entry){ find transition(t,_,e); }` |
| FOL | **WF1-TRA.:** $\forall t, src, trg : transition(t, src, trg) \Rightarrow \texttt{source}(t, src) \wedge \texttt{target}(t, trg)$ <br> **WF1-ITE:** $\forall t, e : ite(t, e) \Rightarrow (\texttt{Entry}(e) \wedge (\exists s : transition(t, s, e))$ <br> **WF2-ITE:** $\forall t, e : (\texttt{Transition}(t) \wedge \texttt{Entry}(e)) \Rightarrow \neg ite(t, e)$ | |

Fig. 10: Mapping VQL patterns and WF constraints

## 5 Evaluation

We conduct several measurements to address the following research questions:

**RQ1**: Which TPTP-compliant theorem provers are most scalable wrt. model size and runtime of model generation?

**RQ2**: How do theorem provers scale compared to other logic solvers for a model generation scenario?

**Target domain:** To address these questions, we perform model generation scenarios and analyze the results in the context of the *Yakindu Statecharts* industrial modeling environment introduced in section 2.1. We use the metamodel shown in Figure 1, which contains 13 classes, including an enum class, and 6 references. Moreover, the Yakindu metamodel covers all mapping rules introduced in section 4. We also formalize 17 WF constraints as graph predicates to further restrict the model generation scope. Finally, we provide an initial instance model as a seed for model generation which contains only a single root node, thus the underlying solvers have full responsibility in model generation. Examples of input and output files as well as our measurement results are on GitHub[4]. Altogether, *Yakindu Statecharts* provide a sufficiently complex case to assess the proposed mapping and the underlying theorem provers, and it has been used as a case study in existing papers of model generation [27,30].

### 5.1 Research Question 1 (RQ1)

**Measurement setup:** We compare the scalability of all TPTP-compliant theorem provers available on the *System on TPTP*[5] website, which is the official TPTP web interface for solving FOL problems for theorem proving competitions. *System on TPTP* lists 73 solvers and 87 different solver configurations that can be called directly on their servers[6] through HTTP requests.

Our experimentation consists of three phases. For all three phases, we generate constraints to avoid containment cycles of up to 5 objects, which is a parameter used in existing research such as [28].

Phase I: As a *preliminary step*, we attempt to generate a small model containing 9-10 nodes within a time limit of 1 minute with each listed TPTP-prover.

[4] https://github.com/ArenBabikian/publication-pages/wiki/Automated-Generation-of-Consistent-Graph-Models-with-Theorem-Provers

[5] http://tptp.cs.miami.edu/cgi-bin/SystemOnTPTP

[6] Intel Xeon CPU E5-4610 2.40GHz, 128GB RAM, Linux 3.10.0

Note that from the 9-10 output nodes, 3 nodes are enforced by the enum mapping, 1 node is defined in the initial model and 5-6 nodes must be generated by the theorem prover. We perform this experimentation three times and we manually analyze the output. If a theorem prover is unable to read the input TPTP problem or is incapable of generating a finite model according to the specifications, it is disqualified for the subsequent two steps of our workflow.

PHASE II: This phase involves *small-size model generation* to further eliminate weak TPTP solvers. For each qualified solver, we generate finite models with increasing size (starting from 5 objects as a lower bound, with a step size of 5 objects). We set a timeout of 1 minute for each generation run. We execute each generation run 10 times and take the median of the execution times of successful runs (i.e. that provide a finite model as result within the given timeout).

We also measure the ratio of failed runs for each model size. We end the sequence of model generations for a given solver if all 10 runs at a same size specification fail to output a finite model. Considering that we are running the measurements on a server, we cannot influence warm-up effects and memory handling. After this second phase, we keep the (four) best performing solvers.

PHASE III: We complete our experimentation by performing *large-scale model generation*. For this phase, we perform the same data collection as for PHASE II. However, we begin model generation at a size of 30 objects and use step size of 10 objects. Furthermore, we use a timeout of 5 minutes and we perform each generation run 20 times.

**Scalability in model size:** We compare model size derived by TPTP solvers.

PHASE I: Among the 87 prover configurations provided on the TPTP server, only 8 configurations were able to generate models with 9-10 objects, namely CVC4 (SAT-1.7), DarwinFM (1.4.5), E-Darwin (1.5), Geo-III (2018C), iProver (SAT-3.0), Paradox (4.0), Vampire (SAT-4.4) and Z3 (4.4.1). The MACE2 (2.2) prover also claimed generating a finite model for the given inputs. However, after manual analysis of the output, no generated finite model was found. As a result, we decided to drop MACE2 from the following measurement phases.

PHASE II: Figure 11a presents the complete measurements for scalability analysis of the 4 least scalable remaining solver configurations. PHASE II results for the 4 more scalable solver configurations are included in Figure 11b, along with their results for PHASE III. Figure 11a contains the median runtime (as provided by the server) of successful model generations wrt the size of the generated model while the runtime required for the mapping itself is excluded (as it is negligible). Measurements for PHASE II are performed for models of up to 25 objects, while measurements for larger models correspond to PHASE III.

Figure 11c presents the ratio of failed model generation runs wrt. model size. When all runs fail in generating models, the failure ratio becomes 1 and no further model generation runs are performed. Notice that solvers CVC4, DarwinFM, E-Darwin and Geo-III are unable to generate models of 30 objects within the 1-minute timeout period, thus they are excluded from further experiments.

PHASE III: Figure 11b shows that iProver and Z3 dominate in terms of scalability. There exists a steady increase in runtime with respect to generated model

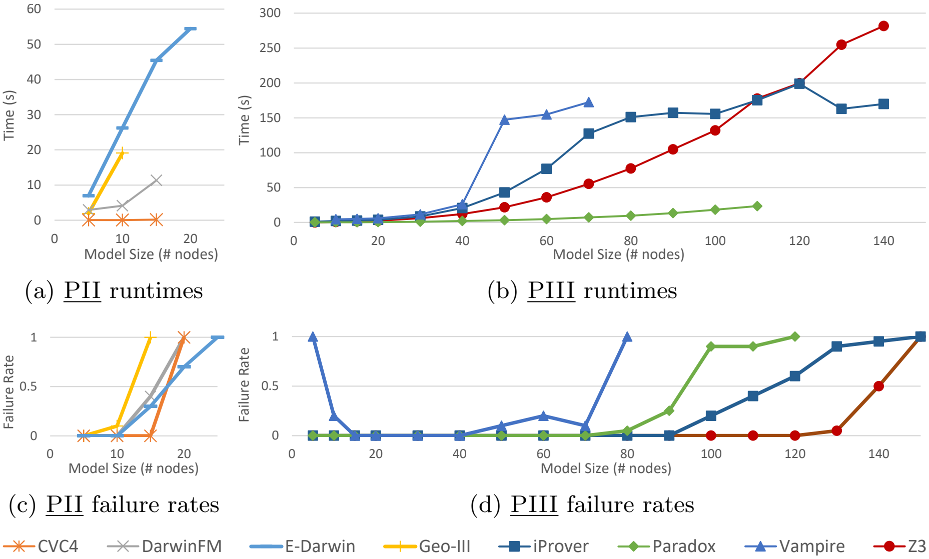

(a) PII runtimes (b) PIII runtimes (c) PII failure rates (d) PIII failure rates

Fig. 11: Results of Phase II and Phase III measurements (incl. failure rates)

size, however, we notice certain inconsistencies when failure rates increase as the generated models become larger. Both solvers can generate models of 140 objects: iProver can do so at a faster rate, however, Z3 does so more consistently with respect to failures. Moreover, it is interesting to see that existing model generation approaches that used Z3 as an underlying solver [28, 32] report inferior results with respect to the size of generated (fully connected) models.

The Paradox solver provides very fast model generation for models of up to 110 objects. Although failure rates are high for large models, by inspecting the measurement data, we notice that Paradox explicitly reports (within timeout) that it is unknown if a model can be generated for the given input.

Scalability of the Vampire solver lacks in comparison to the other solvers. We observe an interesting pattern in failure rates for Vampire: the solver fails often when generating not only large models, but also very small models. In fact, analysis of measurement data shows that in these cases, Vampire states that the input constraints are satisfiable, but it does not generate a finite model. This behavior is similar to that of Paradox, since failures are not caused by timeouts.

**Runtime of solvers:** Runtime differences between solvers are negligible for generated models of size 20 and under. For models larger than 20 nodes, Paradox was the fastest solver as highlighted in Figure 11b. For models with 120 objects or more, iProver is slightly faster than Z3. However, increased failure rates for iProver make the measured median values less reliable than those of Z3.

**RQ1**: *Only 9% (8/87) of theorem prover configurations presented in the* System on TPTP *website are able to generate small models. Only 4 configurations can generate larger models containing 30 nodes. iProver and Z3 are the most scalable provers and are able to generate models of 140 nodes, while Paradox is significantly faster than other solvers for models of up to 110 nodes.*

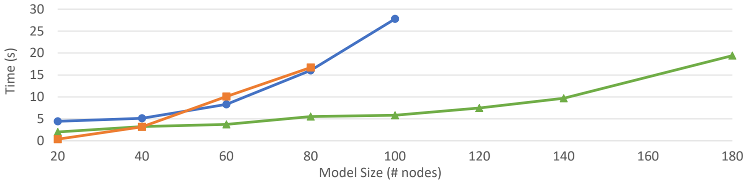

(a) Runtimes for **RQ2** solvers wrt generated model size

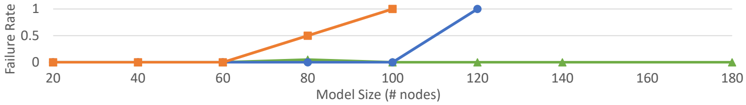

(b) Failure rates for **RQ2** solvers wrt generated model size

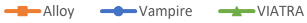

Fig. 12: Results of **RQ2** measurements, including failure rates.

#### 5.2 Research Question 2 (RQ2)

**Measurement setup:** We compare the model generation scalability of the Vampire (4.4) theorem prover to that of two other approaches that use Alloy (4.2) [13] and VIATRA Solver [27, 30] as back-end solvers, respectively. We select Vampire for our experimentation as it is the most scalable theorem prover that we are able to run locally using generated TPTP files as input. We use the most recent stable releases of the solvers to generate graphs of increasing size (starting from models with *exactly* 20 objects, and an increment of *exactly* 20 objects).

We generate constraints to avoid containment cycles of up to 5 objects and we set a timeout of 5 minutes. We execute 20 runs per generated graph size and take the median of the execution times of successful runs (i.e. that provide a finite model as result within the given timeout). To account for warm-up effects and memory handling of the Java 8 VM, we add an extra 5 runs before the actual measurements and call the garbage collector explicitly between runs. We perform measurements on an average personal computer[7] with local installation of solvers. We end the sequence of model generations if none of the 20 runs at a same size specification provide a generated finite model.

**Scalability in model size:** Figure 12a presents the scalability measurements for the Vampire, Alloy and VIATRA solvers. Figure 12b presents the corresponding failure rates. VIATRA was able to generate models of up to 1380 objects, but data points are shown in Figure 12a and Figure 12b for models only up to 180 nodes. We notice that our mapping using the Vampire solver slightly outperforms Alloy, but both approaches are significantly outperformed by the VIATRA-solver, which is coherent with previous research results [30]. The variation in Vampire performance (cf. Figure 11b and Figure 12a) is attributed to the different measurement environments and Vampire versions used to assess each research question.

**RQ2**: *Using Vampire as a back-end solver, our approach scales for 20% larger models with less failures compared to an Alloy-based approach, but it is outperformed by the VIATRA-based approach.*

[7] Intel Core i7-8550U CPU@1.80GHz, 16 GB RAM, Windows 10, Java 1.8, 8 GB Heap

#### 5.3 Threats to validity

**Internal Validity:** The measurements for **RQ1** are performed on a server that acts as a black box with regards to our experimentation. We mitigate this threat by using the same server for the entirety of **RQ1** experimentation. Nevertheless, we take the server runtime output as is for our experimentation. We cannot perform further analysis regarding potential warm-up time and garbage collection, which is mitigated for the experimentation of **RQ2**. Furthermore, we make comparison between our approach and others that use the same back-end solvers (namely, Z3) for model generation. However, we must be aware of the different measurement setups used for each implementation.

**External Validity:** Our approach is limited to a single domain selected based on its past use in related lines of existing research [27, 29, 30, 37]. The domain of Yakindu Statecharts is sufficiently complex to cover all features of our mapping, thus we expect similar scalability results in other domains.

**Construct Validity:** For **RQ1**, we specify a scope ranging from 9 to 10 objects for PHASE I, while we only provide a lower-bound scope specification for the other phases. As for **RQ2**, we ask for an exact number of generated objects. These scope specifications may be disadvantageous for certain solvers (e.g. Alloy, if no upper bound is specified). We mitigate this threat by staying consistent in scope specifications throughout a research question or phase.

### 6 Related work

We provide an overview of various graph generation approaches that derive *consistent* graphs.

**Model generators using back-end logic solvers:** These approaches translate graphs and WF constraints into logic formulae and use a logic solver to generate graphs that satisfy them. EMF2CSP/UML2CSP [8, 10] translates model generation to a constraint programming problem, and solves it by use of an underlying CSP solver. ASMIG [38] uses the Z3 SMT solver [22] to generate typed and attributed graphs with inheritance. An advanced model generation approach is presented in the Formula framework [15] also using the Z3 SMT solver. AutoGraph [26] generates consistent attributed multidimensional graphs by separating the generation of the graph structure and the attributes. Graph generation is driven by a tableau approach, while attribute handling uses the Z3 SMT-solver. [28] proposes a mapping of EMF models enriched with derived features for the formal validation of DSLs. Model generation for this purpose is performed by using Z3 and Alloy as backend solvers.

Logic-solver based generators do ensure consistency and they can also detect inconsistencies in a specification. However, their scalability is comparable to our approach. In fact, we managed to improve scalability of model generation compared to results reported in [28] using Z3 as a back-end solver.

**Custom consistent model generators:** Cartesian genetic programming (CGP) [21] encodes graphs with linear or grid-based genotypes and produces new ones by evolving the initial graph, originally used to produce electronic circuits. Recent work [3, 4] introduces evolving graphs by graph programming, CGP's generalization to arbitrary graphs. However, consistency of models is addressed only on a best-effort basis, i.e. there is no formal guarantee of consistency.

SDG [31] proposes an approach that uses a search-based custom OCL solver to generate synthetic data for statistical testing. Generated models are multi-dimensional and consistent. The study claims scalability by generating a large set of small models. Research in [32] proposes a hybrid approach that uses both a meta-heuristic search-based OCL solver [2] for structural constraints and an SMT solver for attribute constraints, based on the snapshot generator of the USE framework [9]. Generated typed models are (locally) consistent and large, but not fully connected (a large family of small models are generated). The VIATRA graph solver [30] is able to generate large and consistent (fully connected) models by lifting SAT solving algorithms to the level of graphs, and exploiting partial modeling techniques.

Custom approaches are more scalable than our approach, but the inconsistency of a DSL specification cannot be detected, thus, there is no graceful degradation in the case when no consistent models can be derived.

### 7 Conclusion and Future Work

In this paper, we provided a mapping of DSL specifications consisting of an EMF metamodel and well-formedness constraints into first-order logic formulae to be fed into TPTP-compliant theorem provers. As such, we successfully integrated more than 70 different theorem provers for model generation purposes. However, our scalability evaluation of these theorem provers carried out in the scope of an industrial DSL tool revealed that most of those provers cannot be effectively used for model generation purposes – not even for very small models. While these solvers can potentially be efficient in detecting inconsistencies of FOL specifications, our experiments revealed that a different solver profile would be beneficial for model generation purposes despite the similarity in the underlying logic formalization. On the positive side, our mapping improved scalability when using Z3 as a back-end theorem prover for model generation purposes.

As we obtained negative scalability results for the vast majority of theorem provers, we believe that *our case study can serve as an interesting benchmark case for future TPTP competitions* as part of future work. Moreover, we plan to better exploit that theorem provers when no models can exist due to inconsistencies regardless of model size by combining calls to TPTP solvers with custom graph model generation techniques. In this case, TPTP solvers may be able to highlight a minimal set of unsatisfiable elements, which can be checked subsequently during the exploration to prevent inconsistent dead ends.

**Acknowledgements** The first author was partially supported by the Fonds de recherche du Québec - Nature et technologies (FRQNT) B1X scholarship (file number: 272709). This paper is partially supported by MTA-BME Lendület Research Group on Cyber-Physical Systems, and NSERC RGPIN-04573-16 project.
